# Associations between exposure to advertising of foods high in fats, salt and sugar and purchase of energy and nutrients: a cross-sectional study

**DOI:** 10.1017/S1368980024001757

**Published:** 2024-10-10

**Authors:** Amy Heather Finlay, Andrew Jones, Steven Cummins, Amy Yau, Laura Cornelsen, Eric Robinson, Emma Boyland

**Affiliations:** 1 University of Liverpool, Liverpool, UK; 2 Liverpool John Moores University, Liverpool, UK; 3 London School of Hygiene and Tropical Medicine, London, UK

**Keywords:** Food marketing, Foods high in fats, salt and sugar (HFSS), Food purchase, Obesity policy

## Abstract

**Objective:**

To assess associations between self-reported advertising exposure to foods high in fats, salt and sugar and household purchases of energy, nutrients and specific product categories.

**Design:**

A cross-sectional design was used. Advertising exposure data were gathered using a questionnaire administered to the main shopper of each household, and purchase data from supermarkets and other stores for these households were accessed for a 4-week period during February 2019.

**Setting:**

Households in London and the North of England.

**Participants:**

Representative households (*N* 1289) from the Kantar Fast Moving Consumer Goods Panel. Main shoppers were predominantly female (71 %), with a mean age of 54 years (±13).

**Results:**

Linear regression models identified that exposure to foods high in fats, salt and sugar advertising through traditional mediums (including broadcast and print), but not digital, transport, recreational or functional mediums, was associated with greater purchases of energy (9779 kcal; 95 % CI 3515, 16 043), protein (416 g; 95 % CI 161, 671), carbohydrate (1164 g; 95 % CI 368, 1886) and sugar (514 g; 95 % CI 187, 841). Generalised linear models showed that individuals who reported exposure to sugary drink advertising were more likely to purchase sugary drinks (1·16; 95 % CI 2·94, 4·99) but did not purchase more energy or nutrients from sugary drinks. There was no evidence of associations between exposure to advertising for sugary cereals or sweet snacks and purchases from these categories.

**Conclusions:**

There was a strong influence of traditional advertising and sugar-sweetened beverage advertising on household food and drink purchases, thus supporting the need for advertising restrictions across traditional formats and for sugary drinks specifically.

Food advertising is a key aspect of marketing used by the food industry to drive a hierarchy of food promotion effects including awareness, attitudes and purchases of advertised products and brands^(1)^. Reviews and meta-analyses of food marketing research have concluded that foods advertised are often unhealthy^(2)^ and that food advertising is implicated in rising obesity levels^(3)^. There is an abundance of evidence demonstrating the high prevalence of food advertising across a range of media including traditional mediums such as television^(4)^, functional mediums including outdoor signs and outside of schools and stores^(5)^, advertising across transport networks^(6)^ and increasingly across digital media^(7)^. This marketing typically uses powerful creative strategies that further increase the appeal of the marketed brands and products, particularly to children^(2)^. While there are many factors that contribute to weight gain, changes to the environment in recent decades, including increased food marketing, have made weight gain a natural response to an increasingly obesogenic environment^(8)^.

A recent global evidence review and meta-analysis found significant effects of food marketing (television, digital and packaging) on children’s consumption, choice, preference and purchase requests^(9)^. While the majority of food advertising research has explored direct effects on children, adults can also be influenced^(10)^. This is important as adult food purchase decisions not only impact their own consumption but also that of the whole household. Children can also have a substantial impact on parental purchases through pester power in response to food marketing^(11)^. For example, a study conducted in the USA found that over the course of a year, household purchases of child-targeted cereals were thirteen times higher if they were advertised on television, and these purchases were highest in households with one or more children^(12)^.

In 2010, the WHO made limiting the marketing of foods high in fats, salt and sugar (HFSS) to children a priority for Member States^(13)^, due to the overwhelming evidence of negative consequences for health. Only a limited number of countries have since imposed such restrictions, and a majority of these are limited in scope such as only restricting advertising on television and in content specifically designed for children^(14)^. In 2007, the UK government introduced restrictions for HFSS food marketing on children’s television channels and around child-targeted programmes. However, these restrictions did not reduce children’s exposure to food marketing on television despite adherence to restrictions^(15)^. For children aged 4–15 years, exposure to HFSS advertising as a proportion of all food advertising increased post-restrictions, while exposure to HFSS advertising as a proportion of all advertising remained the same^(15)^. In order to sufficiently reduce children’s exposure to unhealthy food advertising, further restrictions in the form of a 9 pm watershed have now been announced^(16)^. In Chile, similar restrictions were implemented in 2016, whereby adverts on television for ‘high-in’ foods were banned around child-targeted programmes and programmes where at least 20 % of the audience are under 14 years. Research identified that these restrictions reduced children’s minutes of exposure by an average of 44–58 %^(17)^. A systematic review concluded that policies restricting food marketing tend to have desirable or potentially desirable effects, but the certainty of evidence was low for all measured outcomes due to the heterogeneity of the existing research^(14)^. Importantly, it is clear that policies can be used to effectively reduce exposure to food marketing; however, the measurement of impacts is complicated due to the integrated nature of marketing and the simultaneous exposures from multiple media. Advertising campaigns can run across a range of mediums to achieve greater exposure and reach of their messages. Additionally, there has been an increase in targeting of specific consumers through digital media. For example, advertising through video game live streaming^(18)^ is growing as brands seek to tap into the lucrative adolescent and young adult market^(18)^.

There is evidence that mandatory policies to reduce exposure to less healthy food advertising have been successful in influencing behaviour^(14)^. This includes advertising policies at the local level; for example, reduced purchases of unhealthy food have been observed following a ban on advertising of HFSS foods across transport networks in London^(19)^. This ban reduced relative energy purchases by 6·7 % and sugar purchases by 10·5 %^(19)^. Similarly, decreases in fast-food purchases by French-speaking households were observed following an advertising ban on fast food in print and electronic media in Quebec, Canada^(20)^. The above examples of policy impact suggest a level of specificity (i.e. the changes in purchase behaviour were in relation to the types of products banned by the policies); however, there is some evidence that advertising operates at both a category and brand level^(10,21)^. This study will explore that further, by examining purchases at a nutrient level (e.g. purchase of fat, protein, sugar, carbohydrate) to capture potential effects of advertising beyond individual product purchases.

Limited research to date has examined the influence of food advertising on energy and nutrient purchases, but considering purchases at this level will enable a greater understanding of the nuance of how advertising may be associated with dietary behaviours and resultant dietary quality. There is also limited research that considers the effect of food advertising on purchase behaviour per household. This is important as household purchases are a useful indicator of consumption. Previous research has identified that household availability of unhealthy foods and soft drinks can predict children’s preference for and intake of these products^(22)^. While this study has particular relevance for UK policy, it is also relevant beyond the UK as globally, there is recognition of the need to protect children from harmful marketing. Further, documenting the relative consumption of energy and nutrients of concern *v*. healthy nutrients is critical to understanding dietary health outcomes^(23)^. Therefore, the main objective of this study was to identify whether there are associations between self-reported exposure to less healthy food marketing across different mediums (traditional, digital, recreational, functional and transport) and household purchases of energy and key nutrients (fat, saturated fat, protein, carbohydrate, sugar, Na, NSP fibre), fruit, vegetable and nut content and household purchase quantity of healthy/less healthy food products (determined by UK Nutrient Profiling Model (NPM)). Secondary objectives were to identify if there are associations between exposure to advertising for specific product categories (sugary drinks, sugary breakfast cereals, sweet snacks) and household purchase of these products and energy and nutrients from that food product category.

## Methods

### Design

A previous study used household purchase data to explore the impact of an HFSS advertising ban across the Transport for London network in 2019^(19)^. Four weeks of baseline household grocery purchases from that study were also used in the present study, alongside questionnaire data administered to the same households over the same 4-week period.

### Participants

Data were from sampled households who are part of the UK Kantar (an international market research company) Fast Moving Consumer Goods panel. Kantar uses quota sampling to recruit households to the panel via email or post. The panel is comprised of approximately 32 000 households and aims to be nationally representative. Households recruited are representative of their region in terms of household size, number of children in the household, socio-economic position and age of main shopper. Households included in the final sample (*n* 1289, representing *n* 3161 individuals) were all located in London and the North of England (North West, North East or Yorkshire and the Humber).

### Data collection

#### Advertising exposure

Questionnaires (Supplementary Material 1) were administered to the main shopper from each recruited household between 10 and 18 February 2019. Questionnaires collected data on main shopper and household characteristics including main shopper sex, age group and BMI, children in the household, adults in the household and region (London or the North of England). Participants reported their employment status, and all main shoppers were coded as being employed (1) or unemployed (0). Socio-economic position was scored according to the National Readership Survey and categorised into three groups; AB (high: upper middle class/middle class), C1C2 (medium: lower middle class/skilled working class) and DE (low: working class/non-working).

Participants reported their exposure to HFSS food and beverage advertising (defined in the questionnaire as: ‘*processed foods high in salt, sugar and fat are those such as sugary drinks, meals from fast food chains, ready meals, sit down meals, sugary breakfast cereals, sweet snacks (e.g. chocolate bars, sweets, cookies/biscuits), savoury snacks (e.g. crisps, salted/flavoured nuts) and desserts (cakes, ice-cream and flavoured yoghurts)*’. All definitions of product categories were adapted from the International Food Policy Study^(24)^. Participants responded to a number of questions investigating their advertising exposure for the previous week. For example, participants were asked how often they had seen advertisements for a range of HFSS products (e.g. sugary drinks) and asked to respond with one of the following answers: ‘I haven’t seen or heard any advertisements’, ‘once’, ‘a few times’, ‘everyday’ or ‘more than once a day’. Definitions for these categories can be found in Supplementary Material 1. Participants were then asked to report (Y/N) if they had seen advertisements for HFSS foods in a range of different settings. Questions covered all mediums classed as traditional, digital, functional, recreational and transport, described to participants as shown in Table 1. These are the same advertising categories used in previous research^(25)^. The survey response rate was 71 %. The percentage of households recording no purchases varied week by week. As there was no clear pattern, any households with no purchase data for the 4-week period were assumed to be random and excluded from the study. Further information on the development of the advertising exposure scale is available in published work^(25)^.

**Table 1:** Categorisation of advertising mediums, adapted from^(25)^

Advertising category	Included mediums
Traditional	Television, radio, text message, newspaper/magazine, email and leaflet
Digital	Online/internet, mobile app, video game and social media
Functional	Billboard/outdoor signs, telephone boxes, school/college/university, signs or displays in supermarket/convenience stores/restaurants, delivery drivers, doctor’s surgery, shopping centre and motorway services
Recreational	Film/cinema, leisure centre/gym/community centre, sports event/concert/community event, giveaway/sample/special offer and pub
Transport	Outside/inside buses, outside/inside tube, tram or train, outside/inside of tube or train station, bus stop, taxi and back of bus ticket

#### Household nutrient purchase

Participants used barcode scanners to record food and beverage purchases brought back to their homes from supermarkets, corner shops and any other out-of-home settings. Non-barcode products (e.g. loose fruit and veg) were recorded using bespoke barcodes. Participants were additionally required to provide price information from receipts. Once scanned, purchases were matched to existing nutritional data. Kantar collects nutritional data through direct measurement in outlets twice a year and through the use of product images provided by Brandbank. Regular data collection helps to capture product reformulation. Due to the nutritional data being collected in real time, researchers were unable to double-code the nutritional content of food purchases. However, Kantar employs extensive automatic processes using machine learning to detect and counter potentially suspicious activity or fraud. Where nutritional information was not available, values were copied from similar products, or average values for the category or product type were calculated. For this study, take-home purchase data for a 4-week period from 4 February to 3 March 2019 were analysed to coincide with when the advertising exposure questionnaire was completed. Self-reported sociodemographic data relating to the main shopper and household characteristics are collected annually by Kantar from the panellists and were included with the purchase data. Purchased foods were classified as healthy or less healthy by the UK NPM^(26)^. UK NPM scores are calculated by considering the nutrients and food components of the product. This measure combines scores (maximum of ten for each component) for negative food components exceeding specified thresholds (i.e. energy, sugar, fat, Na) and subtracts from the score if products exceed thresholds required for positive components (protein, fibre, fruit, vegetable and nut content). For food products, a total score of 4 and above classifies a product as less healthy. Drinks are classified as less healthy if they score 1 or higher. The fruit, vegetable and nut content of purchased foods were estimated for market categories so do not have the same accuracy per product as nutrient data. To determine these scores, categories were assigned values of 0 (<40 % fruit, vegetable or nut content), 1 (40–60 % fruit, vegetable or nut content) or 5 (>80 % fruit, vegetable or nut content). The UK NPM was used to categorise foods as it has direct policy relevance in the UK. This profiling model is currently used to determine which products can and cannot be advertised on television to children and where restrictions exist elsewhere (e.g. across TfL networks).

### Analysis

Based on the survey responses, participants were binary coded as exposed to HFSS advertising through each media or not and exposed to advertising for specific food types or not (sugary beverages, sugary cereals, sweet snacks). Purchases were combined for each household, with the total sum calculated for purchased energy (kcal), fat (grams), saturated fat (grams), carbohydrates (grams), fibre (grams), protein (grams), Na (grams) and sugar (grams). For our analyses, for each household means were calculated for the fruit, vegetable and nut content of purchased food and the proportion of purchases classed as less healthy.

Multiple linear regressions with robust standard errors were performed to assess whether food advertising overall and across various mediums was associated with household purchases of energy, nutrients, fruit, vegetable and nut content and healthiness of purchased foods. Multiple linear regressions were used as they allowed for the exploration of the linear relationship between food marketing and nutrient purchases alongside a number of other predictor variables. Generalised linear models explored associations between exposure to advertising by product category (sugary beverages, sugary cereals and sweet snacks) and the likelihood of purchase of products from that category. Generalised linear models were deemed appropriate for this analysis as the outcome variable was binary. Linear models with robust standard errors assessed energy and nutrients purchased from advertised product categories. All models were adjusted for main shopper sex, age group and employment status as well as number of children in the household, number of adults in the household, socio-economic position and region (London or the North of England). Models were not adjusted for main shopper BMI, as there was a high number of missing values for this variable (*N* 235). For all models, the largest variance inflation factor was 1·40, so any effects of (multi)collinearity were minimal.

Heteroscedasticity was detected through visual observation of residual plots and confirmed using the ‘check_heteroscedasticity’ function in R (Performance package, version 0·9·2). This function conducts a Breusch–Pagan test^(27)^ and indicates that heteroscedasticity is present in the model if *P* < 0·05. The observed heteroscedasticity was due to a number of high-leverage data points. To account for this, linear models were conducted with robust standard errors to reduce any potential bias and improve statistical inferences. To adjust for multiple comparisons, the *p* value was divided by the number of models (*n* 10); therefore, results were judged as significant at *P* < 0·005. Analyses were conducted in R, with packages, ‘estimatr’ version 1.1.0^(28)^ to conduct robust linear models, ‘performance’ version 0.9.2^(29)^ to assess the performance of regression models and ‘marginaleffects’ version 0.7.0^(30)^ to estimate marginal effects of generalised linear models.

## Results

### Demographics

1289 households completed the advertising survey and recorded food purchases for the 4-week period in February 2019. The majority of household main shoppers were female (71·37 %, *n* 920), currently working (63·69 %) with a mean age of 53·81 (±13·38) and a mean BMI of 27·36 kg/m^2^ (±5·71). The majority of households had no children (72·46 %) and were in the middle socio-economic group (i.e. classed as C1 or C2 by the UK Office for National Statistics; 60·28 %)^(31)^. Included households purchased *n* 143 720 items over the study period, of which 37·2 % (*n* 53 469) were classed as less healthy. A summary of the main shopper and household characteristics is provided in Table 2.

**Table 2:** Sociodemographic characteristics of participants (*n* 1289 households)

Sociodemographic characteristics	Categorisation	*N*	%
Sex	Female	920	71·37
	Male	369	28·63
BMI*	Underweight	25	1·94
	Normal	394	30·57
	Overweight	355	27·54
	Obesity	280	21·72
	Missing	235	18·23
Age group	18–34	141	10·94
	35–44	235	18·23
	45–54	344	26·69
	55–64	300	23·27
	≥65	269	20·87
Household size	1	268	20·79
	2	477	37·01
	3	237	18·39
	4+	307	23·82
Children† in household	No	934	72·46
	Yes	355	27·54
Region	London	562	43·60
	North of England	727	56·40
SEP‡	AB	282	21·88
	C1C2	777	60·28
	DE	230	17·84
Working status§	Not working	465	36·07
	Working	821	63·69
	Missing	3	0·23

* BMI was calculated using self-reported height and weight data. 18.23 % of participants did not provide this data. The remaining participants were categorised as having underweight (BMI < 18.5 kg/m^2^), healthy weight (BMI ≥ 18.5 and <25 kg/m^2^), overweight (BMI ≥ 25 and <30 kg/m^2^) or obesity (BMI ≥ 30 kg/m^2^).

† Household members under the age of 16 were classed as children.

‡ Socio-economic position (SEP) classifications were based on the National Readership Survey occupational social grade classification (A, B, C1, C2, D, E). We categorised these into three SEP groups: high (AB), middle (C1C2) and low (DE) as per ref^(25)^.

§ Not working: on a government-sponsored training scheme, retired, a student, looking after home or family, long-term sick or disabled, actively looking for paid work, unemployed and not looking for work. Working: full-time employee, part-time employee, self-employed or freelance, working for your own or family’s business, away from work, doing any other kind of paid work.

### Advertisement exposure

Table 3 summarises exposure data. The largest proportion of main shoppers reported exposure to traditional advertising (73·70 %) followed by functional (50·81 %) and digital advertising (37·55 %) (Table 3), and the most frequent food category (of those measured) that participants reported exposure to across any advertising medium was sweet snacks (54·85 %).

**Table 3:** Self-Reported advertising exposures (*n* 1289 main shoppers of included households)

Category	Advertising type	Exposures (freq.)	%
Exposure	*Traditional*	950	73·70 %
	*Functional*	655	50·81 %
	*Digital*	484	37·55 %
	*Transport*	447	34·68 %
	*Recreational*	236	18·31 %
	Product type	Exposures (freq.)	%
	*Sweet snacks*	707	54·85
	*Sugary beverages*	679	52·69
	*Sugary cereals*	533	41·35

Participants were classed as ‘exposed’ or ‘not exposed’ for each medium and food category. Participants were classed as exposed if they had seen any HFSS in the last 7 d across the above mediums and if they had seen any of the specified food categories advertised across any medium in the last 7 d.

Table 4 shows the means and standard deviations of purchased energy and nutrients, as well as the mean fruit, vegetable and nut score, and mean scores for healthiness (according to the UK NPM) over the 4-week study period. Also shown in Table 4 is the number of households who purchased sugary beverages (*n* 1120), sugary cereals (*n* 869) and sweet snacks (*n* 1057).

**Table 4:** Energy and nutrient purchases for the 4-week study period per household

	Categories	Purchases (mean)	sd
Overall purchases	*Energy (kcal)*	102 958·80	56 963·20
	*Fat (g)*	4355·49	2666·91
	*Saturated fat (g)*	1649·84	987·34
	*Carbohydrate (g)*	11 137·79	6691·01
	*Protein (g)*	3996·28	2259·37
	*Fibre (g)*	924·60	534·17
	*Na (g)*	135·07	117·69
	*Sugar (g)*	4603·51	2874·61
	*Fruit, vegetable and nut score* *	1·59	0·25
	*NPM score* **	0·37	0·11
Sugary beverage purchases	*Households (N)*	1120	
	*Energy (kcal)*	1256·41	1837·43
	*Fat (g)*	19·30	53·14
	*Saturated fat (g)*	12·30	24·07
	*Carbohydrate (g)*	230·07	368·66
	*Protein (g)*	27·03	83·48
	*Fibre (g)*	18·25	38·32
	*Na (g)*	1·03	1·64
	*Sugar (g)*	190·46	332·19
	*Fruit, vegetable and nut score*	1	0
	*NPM score*	0·28	0·32
Sugary cereal purchases	*Households (N)*	869	
	*Energy (kcal)*	6746·76	5582·79
	*Fat (g)*	99·63	108·63
	*Saturated fat (g)*	23·62	29·07
	*Carbohydrate (g)*	1231·66	1024·88
	*Protein (g)*	168·16	141·03
	*Fibre (g)*	131·30	122·27
	*Na (g)*	2·75	3·03
	*Sugar (g)*	255·41	274·64
	*Fruit, vegetable and nut score*	2·69	2·06
	*NPM score*	0·34	0·38
Sweet snack purchases	*Households (N)*	1057	
	*Energy (kcal)*	6031·77	5559·86
	*Fat (g)*	259·52	274·66
	*Saturated fat (g)*	131·62	144·55
	*Carbohydrate (g)*	841·03	787·42
	*Protein (g)*	73·59	80·22
	*Fibre (g)*	25·24	27·27
	*Na (g)*	1·36	1·48
	*Sugar (g)*	701·52	660·70
	*Fruit, vegetable and nut score*	7·33	6·34
	*NPM score*	1	0

* Mean fruit, vegetable and nut (FVN) score for all items per household. All items were scored as 0(<40 %FVN), 1(40–60 %FVN) or 5(>80 %FVN).

** Using the UK NPM, all products were classed as healthy (0) or less healthy (1), and the mean score was calculated across all household purchases.

### Associations between food advertising exposure and purchases of energy and nutrients by nutrient categories

Table 5 summarises the main regression models investigating associations between advertising exposures and nutrient purchases, adjusted for main shopper and household characteristics. Unadjusted models are shown in Supplementary Material 2.

**Table 5:** Linear models for HFSS advertising exposures and nutrient purchases (un-adjusted models available in Supplementary Material 2)

Outcome	Variable	Adjusted coeff.	Std error	*P* value*	95 % CI	
					Lower	Upper
Calorie purchase (kcal) (F(161 269) = 27·64, *P* < 0.001), adjusted R^2^ of 0·278.	Intercept	21 733·83	6976·55		8046·99	35 420·67
	Traditional	**9779·22** *	**3192·98**	**0·002**	**3515·12**	**16 043·32**
	Transport	–2250·85	3256·65	0·490	–8639·86	4138·17
	Recreational	–3652·47	4022·39	0·364	–11 543·74	4238·80
	Functional	3111·06	3161·22	0·325	–3090·72	9312·84
	Digital	–2896·19	3368·06	0·390	–9503·76	3711·38
Fat purchase (g) (F(161 269) = 20·57, *P* < 0.001), adjusted R^2^ of 0·227.	Intercept	824·63	351·88		134·30	1514·95
	Traditional	405·61	157·00	0·010	97·60	713·61
	Transport	–53·73	163·56	0·743	–374·62	267·16
	Recreational	–231·22	188·78	0·221	–601·57	139·13
	Functional	122·36	153·81	0·426	–179·40	424·11
	Digital	–105·59	160·14	0·510	–419·76	208·58
Saturated fat purchase (g) (F(161 269) = 19·54, *P* < 0.001, adjusted R^2^ of 0·206.	Intercept	450·12	126·10		202·73	697·50
	Traditional	153·58	59·19	0·010	37·45	269·70
	Transport	–15·94	58·98	0·787	–131·64	99·77
	Recreational	–96·27	69·64	0·167	–232·89	40·36
	Functional	38·69	56·35	0·492	–71·85	149·23
	Digital	–52·92	59·78	0·376	–170·20	64·36
Protein purchase (g) (F(161 269) = 21·23, *P* < 0.001), adjusted R^2^ of 0·224.	Intercept	1037·12	293·40		461·52	1612·52
	Traditional	**416·49** *	**130·04**	**0·001**	**161·37**	**671·61**
	Transport	–2·38	134·62	0·986	–266·47	261·72
	Recreational	–221·83	165·23	0·180	–545·99	102·33
	Functional	100·03	127·58	0·433	–150·25	350·32
	Digital	–158·06	140·52	0·261	–433·74	117·61
Carbohydrate purchase (g) (F(161 269) = 25·23 *P* < 0.001), adjusted R^2^ of 0·273.	Intercept	2245·16	842·68		591·96	3898·37
	Traditional	**1164·04** *	**368·29**	**0·002**	**441·52**	**1886·56**
	Transport	–334·40	372·80	0·370	–1065·77	396·97
	Recreational	–59·11	497·06	0·905	–1034·27	916·04
	Functional	378·18	369·07	0·306	–345·88	1102·24
	Digital	–406·62	391·85	0·300	–1175·36	362·13
Sugar purchase (g) (F(161 269) = 20·03, *P* < 0.001), adjusted R^2^ of 0·206.	Intercept	1463·10	365·33		746·38	2179·82
	Traditional	**514·21** *	**166·56**	**0·002**	**187·45**	**840·96**
	Transport	–206·02	171·65	0·230	–542·77	130·73
	Recreational	–39·63	213·03	0·852	–457·55	378·29
	Functional	262·68	170·73	0·124	–72·26	597·62
	Digital	–211·79	175·55	0·228	–556·20	132·61
Na purchase (g) (F(161 269) = 13·68, *P* < 0.001), adjusted R^2^ of 0·115.	Intercept	33·50	13·92		6·19	60·81
	Traditional	5·00	7·53	0·507	–9·77	19·78
	Transport	–4·62	6·06	0·446	–16·52	7·28
	Recreational	–2·97	7·25	0·682	–17·20	11·26
	Functional	5·82	5·97	0·329	–5·88	17·53
	Digital	–8·94	6·02	0·137	–20·75	2·86
NSP fibre (g) (F(161 269) = 19·74, *P* < 0.001), adjusted R^2^ of 0·223.	Intercept	299·16	69·65		162·53	435·80
	Traditional	44·52	31·98	0·146	–16·22	109·26
	Transport	–11·47	31·83	0·719	–73·92	50·97
	Recreational	–7·59	39·80	0·849	–85·67	70·50
	Functional	10·88	30·69	0·723	–49·33	71·09
	Digital	–59·04	32·15	0·067	–122·10	4·03
Proportion of products classed as less healthy (%) (F(161 269) = 5·14, *P* < 0.001), adjusted R^2^ of 0·044.	Intercept	0·33	0·01		0·30	0·37
	Traditional	0·01	0·01	0·136	–0·00	0·03
	Transport	–0·00	0·01	0·560	–0·02	0·01
	Recreational	–0·01	0·01	0·528	–0·02	0·01
	Functional	0·01	0·01	0·171	–0·00	0·02
	Digital	0·00	0·01	0·855	–0·01	0·02
Fruit, veg and nut content (average score) (F(161 269) = 6·847, *P* < 0.001), adjusted R^2^ of 0·074.	Intercept	1·69	0·04		1·61	1·77
	Traditional	–0·04	0·02	0·018	–0·07	–0·01
	Transport	–0·01	0·02	0·579	–0·04	0·02
	Recreational	0·03	0·02	0·110	–0·01	0·07
	Functional	–0·01	0·02	0·395	–0·04	0·02
	Digital	–0·01	0·02	0·381	–0·04	0·02

* To adjust for multiple testing, we considered results to be significant at *P* = 0.005.

#### Kilocalories

Exposure to traditional food advertising was significantly associated with greater household purchases of energy over the 4-week period (9779 kcal (approximately 2445 kcal a week); a 44 % increase), but this effect was not found for exposure to advertising across transport, recreational, functional or digital mediums. Having a BMI classed as ‘normal’ and being employed was associated with lower purchase of calories, while having more adults in the household, having more children in the household, being in the middle socio-economic group (classed as lower middle class and skilled working class) and having a main shopper over the age of 45 were associated with greater purchase of calories.

#### Fat and saturated fat

Advertising exposure was not associated with household purchases of fat or saturated fat for the 4-week period across any of the advertising mediums. Greater purchases of fat and saturated fat were associated with having a main shopper over the age of 55 and having more adults and more children in the household, while lower purchases of saturated fat were associated with having a main shopper with a BMI classed as ‘normal’ and being employed.

#### Protein

Exposure to traditional advertising was associated with greater household purchases of protein (416 g (approximately 104 g a week); a 40·16 % increase) over the 4-week period, but this effect was not found for exposure to advertising across transport, recreational, functional or digital mediums. Greater purchases of protein were associated with having a main shopper over the age of 45, having more adults in the household and having more children in the household while lower purchases of protein were associated with having a main shopper with a BMI classed as ‘normal’ and living in London.

#### Carbohydrate

Exposure to traditional advertising was associated with greater household purchases of carbohydrates over the 4-week period (1164 g (approximately 291 g a week); a 51·85 % increase) but this effect was not found for digital, functional, recreational or transport advertising. Greater carbohydrate purchases were associated with having a main shopper over the age of 55, having more adults in the household, having more children in the household and being in the middle socio-economic group, while lower carbohydrate purchases were associated with having a BMI classed as ‘normal’, being employed and residing in London.

#### Sugar

Exposure to traditional advertising was significantly associated with greater household purchases of sugar for the 4-week period (514 g (approximately 129 g a week); a 35 % increase), but this was not found for exposure to digital, functional or transport advertising. Greater purchases of sugar were associated with having more children in the household, having more adults in the household and being in the middle socio-economic group, while lower purchases of sugar were associated with having a BMI classed as ‘normal’, being employed and residing in London.

#### Sodium

Advertising exposure was not associated with household purchases of Na for the 4-week period across any of the advertising mediums. Greater purchases of Na were associated with having more adults in the household, having more children in the household and being in the middle socio-economic group, while lower purchases of Na were associated with having a BMI classed as ‘normal’.

#### Fibre

Advertising exposure was not associated with household purchases of NSP fibre for the 4-week period across any of the advertising mediums. Greater purchases of fibre were associated with having more adults in the household, having more children in the household and having a main shopper over the age of 45.

#### Fruit, vegetable and nut content

No advertising exposures were associated with the average fruit, vegetable and nut score of purchased products for the households over the 4-week period. Greater fruit, vegetable and nut content of purchased foods was associated with an ‘underweight’ or ‘normal’ BMI and residing in London, while lower fruit, vegetable and nut content of purchased foods was associated with having more children in the household or being in the middle or lower socio-economic group.

#### Food advertising exposure on overall healthiness of purchased foods

No association was observed between exposure to HFSS advertising across any format and the proportion of household purchases that were classed as less healthy. A greater proportion of less healthy foods purchased (and so a smaller proportion of healthy foods purchased) was associated with having more children in the household and being in the lower or middle socio-economic group, while having a smaller proportion of less healthy food purchases was associated with living in London.

#### Food advertising exposure by specific category: energy and nutrient purchase from the category

Exposure to sugary drink advertising across any medium was significantly associated with a greater likelihood of sugary drink purchase (log odds: 3·81, *P* < 0·001). A summary of findings relating to specific product categories is shown in Table 6. However, of those who purchased sugary drinks, advertising exposure was not associated with nutrient purchases from soft drinks. Exposure to sugary breakfast cereal and sweet snack advertising was not associated with the likelihood of purchase from these product categories or purchase of energy or nutrients from these categories. Unadjusted and adjusted models summarising exposure and purchase for specific food groups are shown in Supplementary Material 3.

**Table 6:** Models summarising exposure to advertising for specific food groups and likelihood of purchase from these food groups

Outcome	Intercept	Coeff.	Std error	*P* value	95 % CI	Marginal effect	Std error	*P* value	95 % CI
Exposure to sugary drink advertising on purchase of sugary drinks	**1·16**	**3·81**	**0·51**	**<0·001**	**2·94, 4·99**	**0·39**	**0·05**	**<0·001**	**0·29, 0·50**
Exposure to sugary cereal advertising on purchase of sugary cereals	–0·21	18·37	334·61	0·956	233·31, 182·00	3·03	55·20	0·956	–105·16, 111·22
Exposure to sweet snack advertising on purchase of sweet snacks	0·02	18·92	431·27	0·965	318·09, 332·05	2·11	48·18	0·965	–92·32, 96·55

## Discussion

This study explored associations between household main shopper self-reported exposure to HFSS advertising and household purchases of energy and key nutrients from a large sample of UK households. Findings showed that exposure to traditional advertising (including broadcast, print, text message and email advertising) was associated with greater purchases of energy and nutrients (energy, protein, carbohydrates and sugar). This was not the case for other advertising mediums. In support of this, a study in 2015 compared traditional (TV and print) with online advertising and found that traditional advertising had a greater influence on attention and persuasiveness^(32)^ as measured by questionnaires. Traditional advertising also led to improved attitudes towards the brand compared with online advertising, which is a key predictor of purchase intention^(32)^. This may help to explain the strong observed relationship with traditional advertising in the present study.

It is possible that traditional advertising demands more attention from the consumer than other mediums. Evidence suggests that the impacts of food marketing are stronger with increased perceptual fluency^(33)^. Perceptual fluency may be increased through repeated exposure or through conscious processing of the marketing^(33)^. In the present study, due to the use of binary self-reported measures, we were unable to consider the effects of prolonged or recurrent exposure to HFSS marketing on purchases. As associations between traditional HFSS marketing exposure and household purchases were observed, it could be speculated that greater perceptual fluency occurs in response to food marketing on traditional media as opposed to other formats (i.e. digital, recreational, functional and transport) because greater attention is required and therefore a greater depth of processing may occur.

Over recent years, digital advertising has adapted, becoming more sophisticated and personalised, often encouraging interaction, making it an increasingly powerful form of marketing^(34)^. However, in the present study, exposure to digital advertising was not associated with purchases of any nutrients. It is seemingly more difficult for consumers, particularly children, to distinguish between advertising and entertainment in a digital setting^(34)^, and so it is possible that this advertising was less acknowledged by participants than traditional mediums, and so the self-reported frequency of exposure was underestimated. Similarly, much of the media classed as functional, recreational and transport can be grouped as ‘out-of-home’ advertising, which is typically encountered by an individual on the move or when they are otherwise occupied. It may be expected that this would lead to less direct attention being paid to the advertising, leading to a reduction in reported exposure. Previous research has shown the impacts of digital marketing on the intended use and consumption of unhealthy commodities^(35)^, and more recent research has shown evidence that outdoor food marketing is associated with craving^(36)^. Therefore, further research examining how food marketing is processed by consumers across different formats and the resultant impacts on food purchase and consumption would be informative.

Data from the present study suggest an average household increase in purchases of 9779 kcal, 416 g of protein, 1164 g of carbohydrates and 514 g of sugar over the 4-week period per household for those with a main shopper exposed to traditional HFSS advertising. These findings support actions to further restrict HFSS advertising on television in the UK. This is further warranted by research showing that after initial advertising restrictions to children’s television programming in the UK, exposure to HFSS advertising did not decrease^(15)^. It was determined that children are frequently exposed to advertisements from other TV programming. A global review of food marketing policy^(14)^ found that policies were more likely to be associated with positive outcomes if they were mandatory, if they applied to television advertising, if a nutrient profiling model was used to classify foods and if they were designed to restrict marketing to children over 12 years (in addition to below 12 years). This stresses the need for implemented policies to be thorough and mandatory to achieve optimal outcomes. The television watershed proposed in the UK permits no HFSS advertising before 9 pm^(37)^. This policy is both thorough and mandatory and so would likely have positive impacts on food-related behaviours. A modelling study estimated the potential impact of the HFSS watershed and found that this policy could have a meaningful impact on childhood obesity^(38)^. Positive impacts would likely persist even if advertising is displaced as opposed to removed completely. Previous research assessing the impact of HFSS advertising restrictions across the Transport for London network^(19)^ found that following restrictions, average weekly household purchases were reduced by 1001 kcal, 50·7 g of fat and 80·7 g of sugar. Based on the associations observed in the present study, a total ban on television advertising for HFSS foods could have a significant influence on unhealthy household food purchases.

Greater purchases of protein were also associated with exposure to traditional advertising. While protein is a desirable nutrient, it is unlikely that increased protein in the diet is of great benefit to the majority of UK households because average intakes in the UK population are above recommended levels^(39)^. Purchases of fat, saturated fat, Na and fibre were not predicted by exposure to any advertising, and there was no association observed between advertising exposure and the proportion of household purchases that were classed as less healthy. Fat, saturated fat and Na are frequently high in foods prepared outside of the home. It is possible that if these foods were captured in purchases, associations with these nutrients would have been observed.

Households that reported exposure to sugary drink advertising had a higher likelihood of purchasing sugary drinks over the 4-week period. When just households who purchased sugary drinks were examined, there was no association between exposure to advertising for sugary drinks and energy or nutrients purchased from sugary drinks. This finding is likely due to the high prevalence of beverages with artificial sweetener in place of sugar, which also carry no calories or other nutrients, and the purchase of which would not impact our main outcome variables. While this may suggest that advertising of sugary drinks is associated with purchases of non-sugar alternatives (i.e. a seemingly positive outcome for health), it is important to note that this substitution may not have positive impacts. For example, associations have been observed between artificial sweetener consumption and insulin resistance^(40)^, and there is little evidence that consumption of artificial sweetener as opposed to sugar is associated with weight change^(41)^. Therefore, the presence of artificial sweetener in the diet and its impacts should be considered in future research in order to fully understand the implications of the observed substitutions. Previous research has shown that advertising of sugar-free alternatives to sugary drinks drives the demand for sugary drinks^(42)^. Therefore, it seems that spill-over effects persist in both directions. Specifically advertising of soft drinks is associated with the purchase of soft drinks whether sugar-sweetened or sugar free. This highlights the need for a greater understanding of the wider effects of advertising for specific products, as well as the effects of brand-only marketing (e.g. marketing of a soft drink brand with no specific products), which is currently permitted by a number of food marketing restrictions.

While previous research has confirmed category-level effects of advertising^(21)^, no associations were observed between advertising and the purchase of sugary cereals or sweet snacks. It is possible that advertising for these product types targets children as opposed to adults. The advertising exposure questionnaires provided to participants in this study were completed by the household main shopper, so any advertising seen by children in the household would not have been documented. Sugary cereals in particular are often found to target children through their placement on television and the powerful strategies used in marketing. Additionally, this type of advertising is associated with greater sugary cereal consumption in children^(43)^. It is possible that pester power in response to marketing to children could have influenced household purchases rather than the advertising exposure of the main shopper (as was measured). It may be that purchases of snack foods were less likely to be recorded by household main shoppers. Evidence suggests that snack foods in particular are often purchased impulsively^(44)^. If this is the case, such purchases may not have been captured as part of main household grocery purchases. This could also explain the lack of associations with fat, saturated fat and Na that were observed. Further research into advertising for specific food categories and purchase and consumption of these categories is warranted to understand the observed discrepancies between tested product categories. In addition, consideration of associations between exposure and purchase of food prepared outside of the home is necessary, as these foods now form a substantial contribution to the average diet^(45)^.

### Strengths and limitations

This study has several strengths. Primarily, the panel is assessed by Kantar regularly for representativeness, so the purchases from this large sample are likely to be representative of households in London and the North of England, although not generalisable outside of the UK. Additionally, by using the unique perspective of considering nutrients at the household level, we can attempt to ascertain the impact of a household food shop on the dietary behaviour of consumers. Despite this, there are limitations regarding the use of self-reported advertising exposure. It is likely that a significant amount of advertisement exposure is not consciously attended to and self-reported^(46)^. Although self-reported advertising exposure has some validity as a measure^(47)^, exposure reporting is likely to be under-reported and prone to bias. Some research has examined real-time advertising exposure measurement through wearable cameras^(48)^ and screen capture technology^(49)^, which may be useful when attempting to replicate and expand on the present findings in future research. Evidence suggests that weekly grocery shops remain consistent over time, as a result of habitual purchases and brand loyalty^(50)^. While advertising is an important factor in influencing food choices, preferences are formed over a long period of time, and exposure must be prolonged and consistent^(1)^. Due to the nature of exposure data, the extent of repeated exposure to individual advertisements or campaigns was not a factor we were able to measure in this present study; however, further research around this is warranted. Additionally, while grocery purchases provide some insight into household dietary behaviours, without also accounting for purchases of out-of-home foods (i.e. restaurant meals, takeaways, fast food), we cannot assess the impact of advertising on the whole diet, which would be the key indicator of dietary and overall health.

## Conclusion

This study investigated relationships between exposure to HFSS food advertising and household purchases of key dietary nutrients. Our findings indicate there is a strong influence of traditional advertising and sugar-sweetened beverage advertising on household food and drink purchases, thus supporting the need for advertising restrictions across traditional formats and for sugary drinks specifically. The lack of associations for other advertising mediums and other food categories in the present study must be examined further to understand whether any effects occur outside of conscious awareness. Additionally, as out-of-home food is such a big contributor to caloric intake, investigation into the effects of advertising on the purchase of out-of-home foods is warranted.

## Supporting information

Finlay et al. supplementary material 1 Finlay et al. supplementary material

Finlay et al. supplementary material 2 Finlay et al. supplementary material

## Supplementary material

For supplementary material accompanying this paper, visit https://doi.org/10.1017/S1368980024001757.
