# Supplementary material for: Associations between exposure to advertising of foods high in fats, salt and sugar and purchase of energy and nutrients: a cross-sectional study
Source: Public Health Nutr. 2024 Oct 10;27(1):e207. doi: 10.1017/S1368980024001757 (PMC11604320; doi:10.1017/S1368980024001757)
Supplement: Finlay et al. supplementary material 1 — Finlay et al. supplementary material [file S1368980024001757sup001.docx]

***For those living in London**

Q1. In a typical week **how many trips** do you make on Transport for London services?

|  |  | Drop down list 0 -50+ |
| --- | --- | --- |
| 1 | Tube |  |
| 2 | Rail (Overground or TfL rail) |  |
| 3 | Bus |  |
| 4 | Tram |  |
| 5 | DLR |  |
| 6 | Santander Cycle Hire |  |
| 7 | London River Services |  |
| 8 | Emirates Airline |  |

Q2. In a typical week how many trips on Transport for London Services are you **accompanied by your child/children** (aged 16 or under)?

|  |  | Drop down list 0 -50+ |
| --- | --- | --- |
| 1 | Tube |  |
| 2 | Rail (Overground or TfL rail) |  |
| 3 | Bus |  |
| 4 | Tram |  |
| 5 | DLR |  |
| 6 | Santander Cycle Hire |  |
| 7 | London River Services |  |
| 8 | Emirates Airline |  |

Q3. On your typical trip on Transport for London Services (e.g. your commute to work), what is the name of your…

|  |  |
| --- | --- |
| 1 | Start point (tube, bus, tram, DLR, or rail station) |
| 2 | End point (tube, bus, tram, DLR, or rail station) |
| 3 | I don’t use Transport for London Services regularly |

***For those living in the North of England**

Q4. Have you visited London in the last month?

|  |  |  |
| --- | --- | --- |
| 1 | Yes |  |
| 2 | No |  |

Q5. In a typical week **how many trips** do you make on public transport?

|  |  | Drop down list 0 -50+ |
| --- | --- | --- |
| **1** | Tube/ Subway/ Metro |  |
| **2** | Rail |  |
| **3** | Bus |  |
| **4** | Tram |  |
| **5** | Other |  |

Q6. In a typical week how many trips on public transport are you **accompanied by your child/children** (aged 16 or under)?

|  |  | Drop down list 0 -50+ |
| --- | --- | --- |
| **1** | Tube/ Subway/ Metro |  |
| **2** | Rail |  |
| **3** | Bus |  |
| **4** | Tram |  |
| **5** | Other |  |

***For all participants**

Q7. Currently, which of the following best describes you?

|  |  |  |
| --- | --- | --- |
| 1 | Working as a full time employee |  |
| 2 | Working as a part time employee |  |
| 3 | On a government sponsored training scheme |  |
| 4 | Self-employed or freelance |  |
| 5 | Working paid or unpaid for your own or family’s business |  |
| 6 | Away from work ill, on maternity leave, on holiday or temporarily |  |
| 7 | Laid off |  |
| 8 | Doing any other kind of paid work |  |
| 9 | Retired |  |
| 10 | A student |  |
| 11 | Looking after home or family |  |
| 12 | Long term sick or disabled |  |
| 13 | Actively looking for paid work |  |
| 14 | Unemployed and not looking for work |  |
| 15 | None of the above |  |

Q8. In the past 7 days have you bought food or non-alcoholic drinks that were prepared outside of the home, such as at a restaurant, takeaway, fast-food outlet, cafeteria, supermarket ready-meals/ hot food, or vending machine?

|  |  |  |
| --- | --- | --- |
| 1 | Yes |  |
| 2 | No |  |

Q9. (If yes to Q8)

You said you have bought food or non-alcoholic drink prepared outside the home in the past 7 days.

Please indicate how many times you purchased foods or non-alcoholic drinks from each of these places in the past 7 days.

|  |  | Drop down list 0 -50+ |
| --- | --- | --- |
| 1 | Fast food or take-away, i.e. order from a counter, online, or by phone (e.g. Dominos, McDonalds, Fish & Chips, Indian take-away, Chinese take-away) |  |
| 2 | Sandwich shops, delis, cafes or coffee shops (e.g. Pret a Manger, Greggs, Costa Coffee, Subway) |  |
| 3 | Sit-down restaurant or pub with a waiter/waitress (e.g. Nando’s, Pizza Express, Harvester, Wetherspoons) |  |
| 4 | Work or school/university/college canteen (not including fast food chains) |  |
| 5 | Sandwich/Ready-meal from a supermarket (e.g., M&S, Tesco, Asda, Iceland) |  |
| 6 | Burger, chip or kebab van / ‘street food’ market |  |
| 7 | Convenience shop, corner shop, petrol station or pharmacy (e.g 7-Eleven, Boots, Whistle-Stop) |  |
| 8 | Leisure centre, recreation, or entertainment venue |  |
| 9 | Vending machine |  |
| 10 | Other: Please specify |  |

Q10. In the past 7 days have you bought food or non-alcoholic drinks **for your child/children (aged 16 and under)** that were prepared outside of the home, such as at a restaurant, takeaway, fast-food outlet, cafeteria, supermarket ready-meals/ hot food, or vending machine?

|  |  | Single select |
| --- | --- | --- |
| 1 | Yes |  |
| 2 | No |  |

Q11. (If yes to Q10)

You said you have bought food or drink prepared outside the home **for your child/children** in the past 7 days.

Please indicate how many times you purchased foods or non-alcoholic drinks **for your child/children** from each of these places in the past 7 days.

|  |  | Drop down list 0 -50+ |
| --- | --- | --- |
| 1 | Fast food or take-away, i.e. order from a counter, online, or by phone (e.g. Dominos, McDonalds, Fish & Chips, Indian take-away, Chinese take-away) |  |
| 2 | Sandwich shops, delis, cafes or coffee shops (e.g. Pret a Manger, Greggs, Costa Coffee, Subway) |  |
| 3 | Sit-down restaurant or pub with a waiter/waitress (e.g. Nando’s, Pizza Express, Harvester, Wetherspoons) |  |
| 4 | Work or school/university/college canteen (not including fast food chains) |  |
| 5 | Sandwich/Ready-meal from a supermarket (e.g., M&S, Tesco, Asda, Iceland) |  |
| 6 | Burger, chip or kebab van / ‘street food’ market |  |
| 7 | Convenience shop, corner shop, petrol station or pharmacy (e.g 7-Eleven, Boots, Whistle-Stop) |  |
| 8 | Leisure centre, recreation, or entertainment venue |  |
| 9 | Vending machine |  |
| 10 | Other: Please specify |  |

Q12. In the past 7 days, how many times, if at all, did you use the following food delivery apps?

|  |  | Drop down list 0 -50+ |
| --- | --- | --- |
| 1 | UberEats |  |
| 2 | JustEat |  |
| 3 | Deliveroo |  |
| 4 | FoodHub |  |
| 5 | Other: Please specify |  |

Q13. Are you following any kind of special diet? If so, please tick which of the options below best describes your diet

|  |  |  |
| --- | --- | --- |
| 1 | To lose weight |  |
| 2 | To gain weight |  |
| 3 | For medical reasons (e.g. to lower cholesterol or control diabetes) |  |
| 4 | Gluten free |  |
| 5 | Wheat free |  |
| 6 | Dairy free |  |
| 7 | Vegetarian |  |
| 8 | Vegan |  |
| 9 | Other: Please specify |  |
| 10 | I am not on a special diet |  |

Q14. In the past 7 days, how often did you see or hear advertisements for the following?

|  |  | I haven’t seen or heard any advertisements | Once | A few times | Every day | More than once a day |
| --- | --- | --- | --- | --- | --- | --- |
| 1 | Meals from fast-food chains or take-aways (e.g. Dominos, McDonalds, Fish & Chips, Indian take-away, Chinese take-away) |  |  |  |  |  |
| 2 | Meals from sandwich shops, delis, cafes or coffee shops (e.g. Pret a Manger, Greggs, Costa Coffee, Subway) |  |  |  |  |  |
| 3 | Meals from sit-down restaurants or pubs with a waiter/waitress (e.g. Nando’s, Pizza Express, Harvester, Wetherspoons) |  |  |  |  |  |
| 4 | Supermarket ready meals (e.g. Chinese, Thai, Indian) |  |  |  |  |  |
| 5 | Sugary drinks (e.g. fizzy drinks, fruit drinks, sports drinks, energy drinks, diluted cordials/squash, chocolate milk, specialty flavoured hot drinks that have added sugar) |  |  |  |  |  |
| 6 | Sugary breakfast cereals (cereals that contain added sugar such as chocolate or other flavouring/ fillings or are sweetened with frosting or added sugar) |  |  |  |  |  |
| 7 | Sweet Snacks (e.g. chocolate bars, cookies/biscuits, danish pastries, granola bars, sweets or other confectionary) |  |  |  |  |  |
| 8 | Savoury Snacks (e.g. crisps, tortilla chips, salted or flavoured nuts, salted crackers) |  |  |  |  |  |
| 9 | Desserts (e.g. cakes, ice cream, flavoured yoghurts) |  |  |  |  |  |

Q15. **In the last 7 days, have you seen or heard advertisements for processed foods high in salt, sugar and fat in the following places?**

*processed foods high in salt, sugar and fat are those such as sugary drinks, meals from fast food chains, ready meals, sit down meals, sugary breakfast cereals, sweet snacks (e.g chocolate bars, sweets, cookies/biscuits), savoury snacks (e.g. crisps, salted/flavoured nuts) and desserts (cakes, ice-cream and flavoured yoghurts)*

|  |  |  |
| --- | --- | --- |
| 1 | TV |  |
| 2 | Radio |  |
| 3 | Online / internet |  |
| 4 | Mobile app / video game |  |
| 5 | Social media (e.g., Twitter, Facebook, Instagram, Snapchat, YouTube) |  |
| 6 | In a text message |  |
| 7 | Magazine or newspaper |  |
| 8 | Billboard or outdoor signs (e.g., posters) |  |
| 9 | On the outside or inside of buses |  |
| 10 | On outside or inside of carriages on tube/subway/metro, trams and trains |  |
| 11 | At tube/subway/metro stations or train stations |  |
| 12 | At bus stops |  |
| 13 | On telephone boxes |  |
| 14 | In films or in cinemas |  |
| 15 | At school / at college or university |  |
| 16 | Signs or displays in supermarkets, convenience stores or restaurants |  |
| 17 | At a leisure centres, gyms and community centres |  |
| 18 | Sports event, concert or community event |  |
| 19 | Giveaways, samples or special offers |  |
| 20 | Other: Please specify | Fix |
| 21 | I haven’t seen any advertising for these foods in the last 7 days |  |

Q16. In the past 7 days, have you seen any of the following?

|  |  |  |
| --- | --- | --- |
| 1 | Food or drinks promoted using characters or toys from movies or TV (e.g., Star Wars, Disney, Marvel/DC characters) |  |
| 2 | Food or drinks promoted with characters created by food companies (e.g., Freddo, Munch Bunch, Coco Pops Monkey) |  |
| 3 | Celebrity endorsements of restaurants or food/drinks |  |
| 4 | Professional sport teams or sporting events sponsored by restaurants or food/drink companies |  |
| 5 | Children’s/community sports teams sponsored by restaurants or food/drink companies |  |
| 6 | None of the above |  |

Q17. In the past 7 days, has your child/children (aged 16 or under) asked you to buy any food or non-alcoholic drinks because they have seen advertising with…

|  |  |  |
| --- | --- | --- |
| 1 | Food or drinks promoted using characters or toys from movies or TV (e.g., Star Wars, Disney, Marvel/DC characters) |  |
| 2 | Food or drinks promoted with characters created by food companies (e.g., Freddo, Munch Bunch, Coco Pops Monkey) |  |
| 3 | Celebrity endorsements of restaurants or food/drinks |  |
| 4 | Professional sport teams or sporting events sponsored by restaurants or food/drink companies |  |
| 5 | Children’s/community sports teams sponsored by restaurants or food/drink companies |  |
| 6 | None of the above |  |

Q18. In the past 7 days, have you bought your child/children (aged 16 or under) any food or non-alcoholic drinks with…

|  |  |  |
| --- | --- | --- |
| 1 | Food or drinks promoted using characters or toys from movies or TV (e.g., Star Wars, Disney, Marvel/DC characters) |  |
| 2 | Food or drinks promoted with characters created by food companies (e.g., Tony the Tiger, Coco Pops Monkey, Snap, Crackle & Pop) |  |
| 3 | Celebrity endorsements of restaurants or food/drinks |  |
| 4 | Professional sport teams or sporting events sponsored by restaurants or food/drink companies |  |
| 5 | Children’s/community sports teams sponsored by restaurants or food/drink companies |  |
| 6 | None of the above |  |

Q19. In the past 7 days, have you seen or heard any advertisements for **sugary drinks** in the following places?

*SUGARY DRINKS are drinks that contain* ***added sugar****, like* *fizzy drinks, fruit drinks, sports drinks, energy drinks, diluted cordials/squash, chocolate milk, and specialty flavoured hot drinks that have added sugar* *but DO NOT INCLUDE pure fruit juice.*

|  |  |  |
| --- | --- | --- |
| **1** | TV |  |
| **2** | Radio |  |
| **3** | Online / internet |  |
| **4** | Mobile app / video game |  |
| **5** | Social media (e.g., Twitter, Facebook, Instagram, Snapchat, YouTube) |  |
| **6** | In a text message |  |
| **7** | Magazine or newspaper |  |
| **8** | Billboard or outdoor signs (e.g., posters) |  |
| **9** | On the outside or inside of buses |  |
| **10** | On outside or inside of carriages on tube/subway/metro, trams and trains |  |
| **11** | At tube/subway/metro stations or train stations |  |
| **12** | At bus stops |  |
| **13** | On telephone boxes |  |
| **14** | In films or in cinemas |  |
| **15** | At school / at college or university |  |
| **16** | Signs or displays in supermarkets, convenience stores or restaurants |  |
| **17** | At a leisure centres, gyms and community centres |  |
| **18** | Sports event, concert or community event |  |
| **19** | Giveaways, samples or special offers |  |
| **20** | Other: Please specify | Fix |
| **21** | I haven’t seen any marketing for sugary drinks in the past 7 days | Fix, Exclusive |

Q20. In the past 7 days, have you seen or heard any advertisements for **sugary cereals** in the following places?

*SUGARY CEREALS are those that contain* ***added sugar*** *such as chocolate or other flavouring/ fillings e.g Coco Pops, Krave or are sweetened with frosting or added sugar eg Frosties, Sugar Puffs. DO NOT INCLUDE cereals like Weetabix, Ready Brek, Oatmeal/Porridge)*

|  |  |  |
| --- | --- | --- |
| **1** | TV |  |
| **2** | Radio |  |
| **3** | Online / internet |  |
| **4** | Mobile app / video game |  |
| **5** | Social media (e.g., Twitter, Facebook, Instagram, Snapchat, YouTube) |  |
| **6** | In a text message |  |
| **7** | Magazine or newspaper |  |
| **8** | Billboard or outdoor signs (e.g., posters) |  |
| **9** | On the outside or inside of buses |  |
| **10** | On outside or inside of carriages on tube/subway/metro, trams and trains |  |
| **11** | At tube/subway/metro stations or train stations |  |
| **12** | At bus stops |  |
| **13** | On telephone boxes |  |
| **14** | In films or in cinemas |  |
| **15** | At school / at college or university |  |
| **16** | Signs or displays in supermarkets, convenience stores or restaurants |  |
| **17** | At a leisure centres, gyms and community centres |  |
| **18** | Sports event, concert or community event |  |
| **19** | Giveaways, samples or special offers |  |
| **20** | Other: Please specify |  |
| **21** | I haven’t seen any marketing for sugary cereals in the past 7 days |  |

Q21. In the past 7 days, have you seen or heard any advertisements for **sweet snacks** in the following places?

*SWEET SNACKS are foods such as chocolate bars, cookies/biscuits, danish pastries, granola bars, sweets or other confectionary*

|  |  |  |
| --- | --- | --- |
| **1** | TV |  |
| **2** | Radio |  |
| **3** | Online / internet |  |
| **4** | Mobile app / video game |  |
| **5** | Social media (e.g., Twitter, Facebook, Instagram, Snapchat, YouTube) |  |
| **6** | In a text message |  |
| **7** | Magazine or newspaper |  |
| **8** | Billboard or outdoor signs (e.g., posters) |  |
| **9** | On the outside or inside of buses |  |
| **10** | On outside or inside of carriages on tube/subway/metro, trams and trains |  |
| **11** | At tube/subway/metro stations or train stations |  |
| **12** | At bus stops |  |
| **13** | On telephone boxes |  |
| **14** | In films or in cinemas |  |
| **15** | At school / at college or university |  |
| **16** | Signs or displays in supermarkets, convenience stores or restaurants |  |
| **17** | At a leisure centres, gyms and community centres |  |
| **18** | Sports event, concert or community event |  |
| **19** | Giveaways, samples or special offers |  |
| **20** | Other: Please specify |  |
| **21** | I haven’t seen any marketing for sweet snacks in the past 7 days |  |

Q22. In the past 7 days, have you seen or heard any advertisements for **fast food or takeaway meals** in the following places?

*These can be MEALS from fast-food chains or take-aways (e.g., Burger King, Dominos, McDonalds, KFC, fish and chip shops, Indian or Chinese Takeaway)*

|  |  |  |
| --- | --- | --- |
| **1** | TV |  |
| **2** | Radio |  |
| **3** | Online / internet |  |
| **4** | Mobile app / video game |  |
| **5** | Social media (e.g., Twitter, Facebook, Instagram, Snapchat, YouTube) |  |
| **6** | In a text message |  |
| **7** | Magazine or newspaper |  |
| **8** | Billboard or outdoor signs (e.g., posters) |  |
| **9** | On the outside or inside of buses |  |
| **10** | On outside or inside of carriages on tube/subway/metro, trams and trains |  |
| **11** | At tube/subway/metro stations or train stations |  |
| **12** | At bus stops |  |
| **13** | On telephone boxes |  |
| **14** | In films or in cinemas |  |
| **15** | At school / at college or university |  |
| **16** | Signs or displays in supermarkets, convenience stores or restaurants |  |
| **17** | At a leisure centres, gyms and community centres |  |
| **18** | Sports event, concert or community event |  |
| **19** | Giveaways, samples or special offers |  |
| **20** | Other: Please specify |  |
| **21** | I haven’t seen any marketing for fast food or take-aways in the past 7 days |  |

Q23. In the past 7 days, have you seen or heard any advertisements for **food delivery apps** in the following places?

*For example, Uber Eats, Deliveroo, Just Eat, Hungry House, FoodHub.*

|  |  |  |
| --- | --- | --- |
| **1** | TV |  |
| **2** | Radio |  |
| **3** | Online / internet |  |
| **4** | Mobile app / video game |  |
| **5** | Social media (e.g., Twitter, Facebook, Instagram, Snapchat, YouTube) |  |
| **6** | In a text message |  |
| **7** | Magazine or newspaper |  |
| **8** | Billboard or outdoor signs (e.g., posters) |  |
| **9** | On the outside or inside of buses |  |
| **10** | On outside or inside of carriages on tube/subway/metro, trams and trains |  |
| **11** | At tube/subway/metro stations or train stations |  |
| **12** | At bus stops |  |
| **13** | On telephone boxes |  |
| **14** | In films or in cinemas |  |
| **15** | At school / at college or university |  |
| **16** | Signs or displays in supermarkets, convenience stores or restaurants |  |
| **17** | At a leisure centres, gyms and community centres |  |
| **18** | Sports event, concert or community event |  |
| **19** | Giveaways, samples or special offers |  |
| **20** | Other: Please specify |  |
| **21** | I haven’t seen any marketing for food delivery apps in the past 7 days |  |

Q24. In the past 7 days, has your child/children (aged 16 or under) asked you to buy **sugary drinks** because they have seen advertising?

*SUGARY DRINKS are drinks that contain added sugar, like fizzy drinks, fruit drinks, sports drinks, energy drinks, diluted cordials/squash, chocolate milk, and specialty flavoured hot drinks that have added sugar but DO NOT INCLUDE pure fruit juice.*

|  |  |  |
| --- | --- | --- |
| 1 | Yes |  |
| 2 | No |  |

Q25. In the past 7 days, has your child/children (aged 16 or under) asked you to buy **sugary cereals** because they have seen advertising?

*SUGARY CEREALS are those that contain added sugar such as chocolate or other flavouring/ fillings e.g Coco Pops, Krave or are sweetened with frosting or added sugar eg Frosties, Sugar Puffs. DO NOT INCLUDE cereals like Weetabix, Ready Brek, Oatmeal/Porridge.*

|  |  |  |
| --- | --- | --- |
| **1** | Yes |  |
| **2** | No |  |

Q26. In the past 7 days, has your child/children (aged 16 or under) asked you to buy **sweet snacks** because they have seen advertising?

*SWEET SNACKS are foods such as chocolate bars, cookies/biscuits, danish pastries, granola bars, sweets or other confectionary*

|  |  |  |
| --- | --- | --- |
| **1** | Yes |  |
| **2** | No |  |

Q27. In the past 7 days, has your child/children (aged 16 or under) asked you to buy **fast-food or takeaway meals** because they have seen advertising?

*These can be meals from fast-food chains or take-aways (e.g., Burger King, Dominos, McDonalds, KFC, fish and chip shops, Indian or Chinese Takeaway)*

|  |  |  |
| --- | --- | --- |
| **1** | Yes |  |
| **2** | No |  |

Q28. Have you travelled in London with your child/children in the past 7 days?

|  |  |  |
| --- | --- | --- |
| **1** | Yes |  |
| **2** | No |  |

Q29. In the past 7 days, when travelling in London with your child/children (aged 16 or under), how often did you see or hear advertisements for **sugary drinks**?

*SUGARY DRINKS are drinks that contain added sugar, like fizzy drinks, fruit drinks, sports drinks, energy drinks, diluted cordials/squash, chocolate milk, and specialty flavoured hot drinks that have added sugar but DO NOT INCLUDE pure fruit juice.*

|  |  |  |
| --- | --- | --- |
| **1** | Never |  |
| **2** | Once |  |
| **3** | A few times |  |
| **4** | Every day |  |
| **5** | More than once a day |  |

Q30. In the past 7 days, when travelling in London with your child/children (aged 16 or under), how often did you see or hear advertisements for **sugary cereals**?

*SUGARY CEREALS are those that contain added sugar such as chocolate or other flavouring/ fillings e.g Coco Pops, Krave or are sweetened with frosting or added sugar eg Frosties, Sugar Puffs. DO NOT INCLUDE cereals like Weetabix, Ready Brek, Oatmeal/Porridge)*

|  |  |  |
| --- | --- | --- |
| **1** | Never |  |
| **2** | Once |  |
| **3** | A few times |  |
| **4** | Every day |  |
| **5** | More than once a day |  |

Q31. In the past 7 days, when travelling in London with your child/children (aged 16 or under), how often did you see or hear advertisements for **sweet snacks**?

*SWEET SNACKS are things such as chocolate bars, cookies/biscuits, danish pastries, granola bars, sweets or other confectionary*

|  |  |  |
| --- | --- | --- |
| **1** | Never |  |
| **2** | Once |  |
| **3** | A few times |  |
| **4** | Every day |  |
| **5** | More than once a day |  |

Q32. In the past 7 days, when travelling in London with your child/children (aged 16 or under), how often did you see or hear advertisements for **fast-food or takeaway meals**?

*These can be MEALS from places likes Burger King, Dominos, McDonalds, KFC, fish and chip shops, Indian or Chinese Takeaways.*

|  |  |  |
| --- | --- | --- |
| **1** | Never |  |
| **2** | Once |  |
| **3** | A few times |  |
| **4** | Every day |  |
| **5** | More than once a day |  |

Q33. Are you concerned about the amount of marketing for sugary drinks, sweet and savoury snacks, sugary cereals or fast-food that children see?

|  |  |  |
| --- | --- | --- |
| **1** | Not at all concerned |  |
| **2** | A little concerned |  |
| **3** | Somewhat concerned |  |
| **4** | Very concerned |  |

Q34. Are you aware of any initiatives to restrict advertisements of less healthy foods on public transport? If Yes, please write down the name of the initiative(s) you’re aware of

|  |  |  |
| --- | --- | --- |
| **1** | Yes: please specify |  |
| **2** | No |  |
