# Supplementary material for: Associations between exposure to advertising of foods high in fats, salt and sugar and purchase of energy and nutrients: a cross-sectional study
Source: Public Health Nutr. 2024 Oct 10;27(1):e207. doi: 10.1017/S1368980024001757 (PMC11604320; doi:10.1017/S1368980024001757)
Supplement: Finlay et al. supplementary material 2 — Finlay et al. supplementary material [file S1368980024001757sup002.docx]

**Supplementary material 2: Unadjusted models summarising advertising exposure and energy and nutrient purchases, and healthiness of household purchases.**

| **Outcome** | **Variable** | **Coeff.** | **Std. error** | **P value** | **95% CIs** |
| --- | --- | --- | --- | --- | --- |
| **Calorie Purchase**  **(F(5,1283) = 2.711, p=.019), adjusted R^2^of 0.007.** | Intercept | 97789.06 | 3273.77 |  | 91368.49 to 104209.62 |
|  | Traditional | 8991.99 | 3779.74 | .018 | 1576.84 to 16407.13 |
|  | Transport | -7797.79 | 3761.86 | .038 | -15177.85 to -417.72 |
|  | Recreational | -7510.81 | 4558.78. | .100 | -16454.30 to 1432.68 |
|  | Functional | 5916.57 | 3707.34 | .111 | -1356.55 to 13189.68 |
|  | Digital | -1024.24 | 3622.16 | .777 | -8130.25 to 6081.77 |
| **Fat purchase**  **(F(5,1283) = 2.296, p=.043), adjusted R^2^of 0.005.** | Intercept | 4130.75 | 153.45 |  | 3829.71 to 4431.80 |
|  | Traditional | 380.98 | 177.32 | .032 | 33.12 to 728.84 |
|  | Transport | -255.75 | 182.91 | .162 | -614.58 to 103.07 |
|  | Recreational | -395.07 | 207.83 | .058 | -802.80 to 12.66 |
|  | Functional | 242.86 | 172.87 | .160 | -96.28 to 581.99 |
|  | Digital | -49.08 | 168.66 | .771 | -379.96 to 281.81 |
| **Saturated fat purchase**  **(F(5,1283) = 2.666, p=.021), adjusted R^2^of 0.006.** | Intercept | 1575.54 | 58.02 |  | 1461.71 to 1689.36 |
|  | Traditional | 151.33 | 66.33 | .023 | 21.20 to 281.45 |
|  | Transport | -106.21 | 64.77 | .101 | -233.29 to 20.86 |
|  | Recreational | -150.80 | 75.40 | .046 | -298.73 to -2.87 |
|  | Functional | 85.15 | 62.75 | .175 | -37.96 to 208.25 |
|  | Digital | -42.74 | 61.91 | .490 | -164.19 to 78.71 |
| **Protein purchase**  **(F(5,1283) = 2.934, p=.012), adjusted R^2^of 0.007.** | Intercept | 3778.09 | 124.70 |  | 3533.45 to 4022.72 |
|  | Traditional | 390.84* | 146.41 | .008 | 103.60 to 678.08 |
|  | Transport | -206.68 | 151.65 | .173 | -504.20 to 90.83 |
|  | Recreational | -363.61 | 182.10 | .046 | -720.85 to -6.36 |
|  | Functional | 211.09 | 145.82 | .148 | -74.98 to 497.16 |
|  | Digital | -103.53 | 148.25 | .486 | -394.76 to 187.70 |
| **Carbohydrate purchase**  **(F(5,1283) = 2.401, p=.035), adjusted R^2^of 0.006.** | Intercept | 10523.97 | 386.21 |  | 9766.30 to 11281.64 |
|  | Traditional | 1004.57 | 439.14 | .022 | 143.07 to 1866.07 |
|  | Transport | -994.47 | 432.87 | .022 | -1843.68 to -145.25 |
|  | Recreational | -521.88 | 554.21 | .347 | -1609.14 to 565.39 |
|  | Functional | 666.10 | 434.80 | .126 | -186.90 to 1519.10 |
|  | Digital | -65.56 | 421.16 | .876 | -891.80 to 760.69 |
| **Sugar purchase**  **(F(5,1283) = 3.25, p=.006), adjusted R^2^of 0.009.** | Intercept | 4308.86 | 164.71 |  | 3985.73 to 4631.99 |
|  | Traditional | 487.58 | 192.75 | .012 | 109.44 to 865.72 |
|  | Transport | -496.70 | 190.18 | .009 | -869.81 to -123.60 |
|  | Recreational | -195.99 | 230.22 | .395 | -647.63 to 255.65 |
|  | Functional | 383.00 | 190.40 | .044 | 9.47 to 756.53 |
|  | Digital | -136.33 | 183.14 | .457 | -495.63 to 222.96 |
| **Sodium purchase**  **(F(5,1283) = 1.343, p=.244), adjusted R^2^of 0.000.** | Intercept | 131.45 | 7.24 |  | 117.26 to 145.65 |
|  | Traditional | 6.40 | 7.38 | .386 | -8.08 to 20.88 |
|  | Transport | -7.47 | 7.45 | .317 | -22.09 to 7.16 |
|  | Recreational | -11.70 | 9.29 | .208 | -29.93 to 6.53 |
|  | Functional | 10.77 | 6.39 | .092 | -1.77 to 23.31 |
|  | Digital | -4.90 | 7.12 | .492 | -18.86 to 9.07 |
| **NSP Fibre**  **(F(5,1283) = 1.413, p=.217), adjusted R^2^of 0.002.** | Intercept | 923.31 | 31.61 |  | 861.29 to 985.32 |
|  | Traditional | 35.89 | 35.79 | .316 | -34.32 to 106.10 |
|  | Transport | -52.15 | 35.18 | .138 | -121.15 to 16.86 |
|  | Recreational | -42.28 | 43.32 | .329 | -127.26 to 42.70 |
|  | Functional | 34.28 | 34.71 | .324 | -33.82 to 102.38 |
|  | Digital | -44.61 | 33.40 | .182 | -110.14 to 20.93 |
| **Fruit & veg content**  **(F(5,1283) = 2.749, p=.018), adjusted R^2^ of 0.007.** | Intercept | 1.62 | 0.02 |  | 1.59 to 1.65 |
|  | Traditional | -0.04 | 0.02 | .013 | -0.08 to 0.0.01 |
|  | Transport | 0.01 | 0.02 | .451 | -0.02 to 0.05 |
|  | Recreational | 0.03 | 0.02 | .109 | --0.01 to 0.07 |
|  | Functional | -0.01 | 0.02 | .416 | -0.04 to 0.02 |
|  | Digital | -0.02 | 0.02 | .187 | --0.05 to 0.01 |
| **Classed as Healthy/less healthy**  **(F(5,1283) = 4.881, p<.001), adjusted R^2^of 0.016.** | Intercept | 0.36 | 0.01 |  | 0.35 to 0.37 |
|  | Traditional | 0.01 | 0.01 | .113 | -0.00 to 0.03 |
|  | Transport | -0.01 | 0.01 | .126 | -0.03 to 0.00 |
|  | Recreational | -0.01 | 0.01 | .423 | -0.02 to 0.01 |
|  | Functional | 0.01 | 0.01 | .143 | -0.00 to 0.02 |
|  | Digital | 0.01 | 0.01 | .453 | -0.01 to 0.02 |

*Results were considered significant at p<.005

**Supplementary material 3: Unadjusted and adjusted models summarising exposure to advertising for specific food groups and purchase of nutrients from these food groups.**

| Objective | Outcome | Adjusted model | Adjusted Intercept | Adjusted Coeff. | Std. Error | Sig. | 95% CI |
| --- | --- | --- | --- | --- | --- | --- | --- |
| **Exposure to sugary drink advertising on nutrient purchase from soft drinks** | **Energy** | (F(20,1097) = 1565, p<.001), adjusted *R^2^* of 0.035. | 1032.14 | -113.12 | 110.4 | .306 | -329.71 to 103.48 |
|  | **Fat** | (F(20,1097) = 11.31, p<.001), adjusted *R^2^* of 0.008. | 10.28 | 0.37 | 3.17 | .908 | -5.86 to 6.59 |
|  | **Saturated Fat** | (F(20,1097) = 16.07, p=<.001), adjusted *R^2^* of 0.012. | 7.29 | -0.29 | 1.46 | .843 | -3.16 to 2.58 |
|  | **Protein** | (F(20,1097) = 13.62, p<.000), adjusted *R^2^* of 0.007. | 13.86 | -0.15 | 4.94 | .975 | -9.84 to 9.53 |
|  | **Carbohydrate** | (F(20,1097) = 2604, p<.001), adjusted *R^2^* of 0.047. | 212.18 | -31.70 | 22.15 | .153 | 101.64 to 322.72 |
|  | **Sugar** | (F(20,1097) = 3692, p<.001), adjusted *R^2^* of 0.054. | 174.86 | -30.09 | 19.93 | .131 | -69.19 to 9.01 |
|  | **Sodium** | (F(20,1097) = 20.66, p<.001), adjusted *R^2^* of 0.009. | 0.566 | -0.00 | 0.10 | .994 | -0.19 to 0.19 |
|  | **NSP Fibre** | (F(20,1097) = 13.25, p<.001), adjusted *R^2^* of -0.005. | 8.10 | -2.67 | 2.35 | .256 | -7.28 to 1.94 |
| **Exposure to sugary breakfast cereal advertising on nutrient purchase from sugary cereal** | **Energy** | (F(20,846) = 15.66, p<.001), adjusted *R^2^* of 0.094. | 4504.58 | 266.56 | 371.35 | .473 | -462.32 to 995.43 |
|  | **Fat** | (F(19,847) = 3.962, p<.001), adjusted *R^2^* of 0.035. | 72.40 | 3.39 | 7.70 | .660 | -11.73 to 18.50 |
|  | **Saturated Fat** | (F(20,846) = 8.729, p<.001), adjusted *R^2^* of 0.039 | 17.40 | 2.52 | 2.19 | .251 | -1.78 to 6.81 |
|  | **Protein** | (F(20,846) = 7.90, p<.001), adjusted *R^2^* of 0.076. | 115.30 | 5.18 | 9.41 | .582 | -13.28 to 23.64 |
|  | **Carbohydrate** | (F(20,846) = 19.40, p<.001), adjusted *R^2^* of 0.101. | 796.55 | 50.05 | 67.23 | .457 | -81.90 to 182.01 |
|  | **Sugar** | (F(20,846) = 42.38, p<.001), adjusted *R^2^* of 0.078. | 170.48 | 23.10 | 18.73 | .218 | -13.67 to 59.86 |
|  | **Sodium** | (F(20,846) = 35.48, p<.001), adjusted *R^2^* of 0.082. | 1.61 | 0.21 | 0.20 | .294 | -0.18 to 0.61 |
|  | **NSP Fibre** | (F(20,846) = 8.99, p<.001), adjusted *R^2^* of 0.059. | 91.63 | -0.68 | 8.15 | .934 | -16.68 to 15.32 |
| **Exposure to sweet snack advertising on nutrient purchase from sweet snacks** | **Energy** | (F(20,1033) = 292.60, p<.001), adjusted *R^2^* of 0.055. | 3423.59 | 482.55 | 337.44 | .153 | -179.60 to 1144.71 |
|  | **Fat** | (F(20,1033) = 357.90, p<.001), adjusted *R^2^* of 0.046. | 112.50 | 23.87 | 16.67 | .152 | -8.83 to 56.57 |
|  | **Saturated Fat** | (F(20,1033) = 510.40, p<.001), adjusted *R^2^* of -0.059. | 62.57 | 11.48 | 8.82 | .193 | -5.83 to 28.80 |
|  | **Protein** | (F(20,1033) = 219.7, p<.001), adjusted *R^2^* of 0.041. | 29.80 | 6.16 | 4.93 | .212 | -3.52 to 15.85 |
|  | **Carbohydrate** | (F(20,1033) = 200.90, p<.001), adjusted *R^2^* of 0.056. | 563.70 | 65.51 | 47.85 | .171 | -28.38 to 159.40 |
|  | **Sugar** | (F(20,1033) = 292.00, p<.001), adjusted *R^2^* of 0.056. | 452.59 | 59.42 | 39.99 | .138 | -19.04 to 137.88 |
|  | **Sodium** | (F(20,1033) = 135.60, p<.001),  adjusted *R^2^* of 0.056. | 0.72 | 0.12 | 0.09 | .167 | -0.05 to 0.30 |
|  | **NSP Fibre** | (F(20,1033) = 191.30, p<.001), adjusted *R^2^* of 0.026. | 11.11 | 1.53 | 1.69 | .364 | -1.78 to 4.84 |
|  | **Fruit, Veg and Nut content** | (F(20,1033) = 945.30, p<.001), adjusted *R^2^* of 0.040. | 5.35 | 0.46 | 0.39 | .239 | -0.30 to 1.22 |
